# Predicating the Effector Proteins Secreted by *Puccinia triticina* Through Transcriptomic Analysis and Multiple Prediction Approaches

**DOI:** 10.3389/fmicb.2020.538032

**Published:** 2020-09-22

**Authors:** Yue Zhang, Jie Wei, Yue Qi, Jianyuan Li, Raheela Amin, Wenxiang Yang, Daqun Liu

**Affiliations:** ^1^College of Plant Protection, Technological Innovation Center for Biological Control of Crop Diseases and Insect Pests of Hebei Province, Hebei Agricultural University, Baoding, China; ^2^College of Biological Sciences and Engineering, Hebei Xingtai College, Xingtai, China; ^3^Graduate School of Chinese Academy of Agricultural Sciences, Beijing, China

**Keywords:** *Puccinia triticina*, effector proteins, BAX, qRT-PCR, RNA sequencing

## Abstract

Wheat leaf rust caused by *Puccinia triticina* is one of the most common and serious diseases in wheat production. The constantly changing pathogens overcome the plant resistance to *P. triticina*. Plant pathogens secrete effector proteins that alter the structure of the host cell, interfere plant defenses, or modify the physiology of plant cells. Therefore, the identification of effector proteins is critical to reveal the pathogenic mechanism. We used SignalP v4.1, TargetP v1.1, TMHMM v2.0, and EffectorP v2.0 to screen the candidate effector proteins in *P. triticina* isolates – KHTT, JHKT, and THSN. As a result, a total of 635 candidate effector proteins were obtained. Structural analysis showed that effector proteins were small in size (50AA to 422AA) and of diverse sequences, and the conserved sequential elements or clear common elements were not involved, regardless of their secretion from the pathogen to the host. There were 427 candidate effector proteins that contain more than or equal to 4 cysteine residues, and 339 candidate effector proteins contained the known motifs. Sixteen families, 9 domains, and 53 other known functional types were found in 186 candidate effector proteins using the Pfam search. Three novel motifs were found by MEME. Heterogeneous expression system was performed to verify the functions of 30 candidate effectors by inhibiting the programmed cell death (PCD) induced by BAX (the mouse-apoptotic gene elicitor) on *Nicotiana benthamiana*. Hypersensitive response (HR) can be induced by the six effectors in the wheat leaf rust resistance near isogenic lines, and this would be shown by the method of transient expression through *Agrobacterium tumefaciens* infiltration. The quantitative reverse transcription PCR (qRT-PCR) analysis of 14 candidate effector proteins secreted after *P. triticina* inoculation showed that the tested effectors displayed different expression patterns in different stages, suggesting that they may be involved in the wheat–*P. triticina* interaction. The results showed that the prediction of *P. triticina* effector proteins based on transcriptomic analysis and multiple bioinformatics software is effective and more accurate, laying the foundation of revealing the pathogenic mechanism of *Pt* and controlling disease.

## Introduction

Wheat leaf rust caused by *Puccinia triticina* is an airborne, destructive, and obligate biotrophic fungal diseases. Epidemic of this disease can cause 15–45% losses of production and threaten food security worldwide. Plant – pathogen interaction is regulated by multilayers immune mechanism, which can be activated through recognition of pathogens by host-resistant genes. Pathogens interfere with the host defensive identification signal network by secreting effector proteins that reprogram plant cell structure or metabolism (Kamoun, 2006; Lo Presti et al., 2015). The fungal effector proteins can be attached to the cell wall to act in plant apoplast or be transferred to the plant cells targeting specific host proteins or entering corresponding cell tissues (Lo Presti et al., 2015). Understanding the mechanisms of different pathogenic effector proteins can reveal molecular mechanisms of pathogenicity and develop sustainable control strategies for crops (Dalio et al., 2017).

To date, only a few pathogenic fungal effectors have been obtained, and their functions have been understood. However, with the development of whole genome sequencing and the increased numbers of pathogen genomes, complex computational methods have been developed to predict candidate secreted effector proteins (CSEPs) in the pathogen genomes (Sperschneider et al., 2015). Predicting the CSEPs suggests that it may be located extracellularly or belong to the secreted proteins. Many studies have shown that the avirulent genes or effectors encoded by pathogenicity-related genes are secreted proteins. For example, the encoded product of the flax rust fungal *AvrL567* (Dodds et al., 2004; Catanzariti et al., 2006), *Avr1b* of *Phytophthora sojae* (Shan et al., 2004), the avirulence genes of rice blast fungus *Avr Pita* and *Pwl2* (Sweigard et al., 1995), the proteins encoded by *AvrSr35* (Salcedo et al., 2017) and *AvrSr50* (Chen et al., 2017) of *Puccinia gramminis* f. sp. *tritici* are secreted proteins. Numerous researches have shown that elicitors and pathogenic factors secreted out from the pathogen, which can be recognized by plant receptor proteins (D’Silva and Heath, 1997). The role of the signal peptide is to direct the proteins from the cell to the endoplasmic reticulum. It is cleaved by cotranslation, and the mature protein is released from the cell by the Golgi during the secretion (Dalio et al., 2017). Therefore, signal peptide play an extremely important role in the transport of CSEPs and the recognition of plant receptor proteins (Catanzariti et al., 2006). These products encoded by avirulence genes with signal peptide may be involved in the process of information transfer after recognizing plant elicitor receptors (Han et al., 2005). Based on a comprehensive analysis of existing effectors, it was found that most of the CSEPs have the following characteristics, such as containing signal peptide (SP), no transmembrane domain, and small molecular weight (5–30 kDa). In addition, analysis is based on a number of secondary features, such as effector protein motifs, nuclear localization signals, genomic localization, and three-dimensional structures. Based on the characteristics of these effector proteins, models and software have been developed for predicting CSEPs. SignalP (Bendtsen et al., 2004) based on the combination of several artificial neural networks is capable of predicting signal peptide/non-signal peptide; the presence and location of the SP site in the amino acid sequence are different in different organisms, such as Gram-positive/Gram-negative prokaryotes and eukaryotes. TargetP (Emanuelsson et al., 2000) is reported for the prediction of gene localization and is capable of distinguishing chloroplast transit peptide (cTP), mitochondrial transit peptide (mTP), and signal peptide (SP) with higher sensitivity and specificity than any other available subcellular localization prediction software. It has of relatively good capability of the site prediction for relevant target sequences. Predicting the location and the number of transmembrane helices is very important for membrane proteins. TMHMM (Krogh et al., 2001) established by the hidden Markov model has been built to specifically model the variable protein regions of membrane proteins. The hydrophobicity, charge bias, and helix length are combined into one model through setting the upper and lower limits of membrane helix length to predict membrane proteins and to count the different amounts of topologies. Up to now, bioinformatics analysis has been applied to predict fungal effectors in *Fusarium graminearum*, *Puccinia striiformis* f. sp. *tritici*, etc. (Prasad et al., 2019).

Recent progress in genomics results in many high-quality *P. triticina* genomes and gene expression profiles during wheat infection by *P. triticina* (Bruce et al., 2014; Cuomo et al., 2017). The expression profiles of secreted proteins during the interaction between wheat and *P. triticina* are typically complicated. There are many secreted non-effectors that play roles in colonization and protection of *P. triticina* from competing microbes, differentiation of fungal structures, and cell-to-cell communication. Therefore, accurate mining of effector proteins is time saving for subsequent experimental validation. Using machine learning approach to predict effector proteins is the most convenient and effective way (Sperschneider et al., 2018a).

EffectorP developed based on the Bayesian classification can improve the prediction efficiency and precisely screen the effectors secreted by pathogenic fungi according to the molecular weight, the net charge, and the percentage of cysteine, serine, and tryptophan of the protein (Sperschneider et al., 2016).

Transcriptome analysis is conducive to identify the CSEPs and provide a tool for studying the interaction between rust and host and discovering the pathogenesis of rust. Because of the large genome and complex structure of rust, RNA-seq is an effective method for the CSEPs’ identification. At present, this approach has been applied to different species and genera of rust.

*P. triticina* is a dikaryotic fungus in the most time of life cycle. The genome of *P. triticina* is around 135 MB, which is the largest one among *P. gramminis* f. sp. *tritici*, *P. striiformis* f. sp. *tritici*, and *P. triticina*. A total of 1,358 secreted proteins were predicted based on the genome and RNA-seq of *P. triticina* isolate – BBBD. There were 784 candidate effector proteins expressed in diverse stages of life cycle including the sexual stage (Cuomo et al., 2017). Different pathogenic types exhibit different CSEPs when interacting with the host. We have carried out RNA-seq for three different *P. triticina* pathotypes (THSN, JHKT, and KHHT) at the stage of dormant urediniospores, germinated urediniospores, and interaction with wheat cultivar “Thatcher” at 6 days post-inoculation (dpi).

In this study, we used many software including SignalP v4.1, TargetP v1.1, TMHMM v2.0, and EffectorP v2.0 to predict the effector proteins based on RNA-seq data from three *P. triticina* isolates at three different stages. The candidate effector proteins were verified by *A. tumefaciens*-mediated transient expression assay and qRT-PCR. The results will lay a foundation for further study on effector proteins and their pathogenesis of *P. triticina*.

## Materials and Methods

### Plant Materials and Fungal Isolates

Wheat cultivars Thatcher, 36 near-isogenic lines or cultivars [TcLr1, TcLr2a, TcLr2b, TcLr2c, TcLr3, TcLr3bg, TcLr3ka, TcLr9, TcLr10, TcLr11, TcLr14a, TcLr14b, TcLr15, TcLr16, TcLr17, TcLr18, TcLr19, TcLr21, TcLr23, TcLr24, TcLr25, TcLr26, TcLr28, TcLr29, TcLr30, TcLr32, TcLr33, TcLr36, TcLr38, TcLr39, KS91WGRC11 (*Lr42*), TcLr44, TcLr45, Pavin76 (*Lr46*), TcLr47, TcLr50] and Zhengzhou 5389, *P. triticina* isolate THSN, which is compatible interacted with “Thatcher” and Zhengzhou 5389, and *N. benthamiana* were used in this study. Wheat and *N. benthamiana* plants were grown in the greenhouse at 23 and 16°C, respectively. The THSN was inoculated on the wheat cultivar Zhengzhou 5389 and incubated in the dark at 20°C with 100% relative humidity for 14 h. Plants were then transferred to a greenhouse with 16/8 h day/night cycle at 23–25°C.

### RNA Preparation and RNA-Seq

The *P. triticina* pathotypes were purified by isolating single uredinium and propagated in Zhengzhou 5389, and then, the total RNA of *P. triticina* at dormant urediniospores, germinated urediniospores, and the same position of the inoculated “Thatcher” leaf tissue by *P. triticina* isolates KHTT, JHKT, and THSN at 6 dpi were isolated using RNA extraction kit (RNeasy FFPE Kit, Qiagen) following the instructions, respectively (with two or three biological replicates). The Illumina HiSeq 2000 sequencing platform was used for sequencing total RNA after testing the quality of the extracted RNA (conducted by BGI Company, China).

### Plasmid Construction and Preparation

The primers used for plasmid construction are shown in Supplementary Table S1. Primers were designed based on the complementary DNA (cDNA) sequence containing the longest open reading frame (ORF) of the CSEPs. Thirty candidate genes screened from the RNA-seq data were PCR amplified and cloned using FastPfu DNA polymerase (TransGen Biotech, Beijing, China). To express the candidate effector genes in *N. benthamiana*, the sequences encoding mature proteins without the putative signal peptide (SP) were recombined into the PVX vector pGR107. *A. tumefaciens* strain GV3101 cultured in Luria–Bertani (LB) media containing 50 mg/ml of rifampicin at 28°C with shaking at 220 rpm for 48 h was used for transient expression in *N. benthamiana*. To detect the expression levels of genes, all primers (Supplementary Table S2) were designed using Primer 5 based on the open reading frame from the RNA-seq.

### The Identification of the Coding Sequences

Clusters and unigenes from the RNA-seq data of *P. triticina* isolates (KHTT, JHKT, and THSN) were analyzed using TransDecoder software to identify the candidate coding sequence (CDS). The homologous sequences predicted by Pfam were searched by Blast alignment SwissProt database and Hmmscan. Then, the CDS were used to predict one by one according to the order of SignalP v4.1, TargetP v1.1, TMHMM v2.0, and EffectorP v2.0 (Supplementary Figure S1).

### Signal Peptide Prediction

SP cleavage site prediction was performed on the CDS sequences using the SignalP v4.1 server^1^. Sequence submission and prediction approaches were based on the description (Nielsen, 2017). The results showing “YES” of D score, which mean the protein contains an N-terminal SP sequence, were selected.

### Subcellular Localization Prediction

A new fasta file containing the sequences predicted by SignalP v4.1 was generated and input to the TargetP v1.1 server^2^ for cell localization prediction. Inputting sequences were according to the instruction (Emanuelsson et al., 2000). The credibility partition is determined by the RC value, which is the difference score (diff) of two consecutive highest values; the higher the RC value level is, the lower the confidence of the data is. This study retained data located at “S” (containing SP) with RC values of 1, 2, and 3, and “M” (containing mitochondrial transit peptide mTP) with RC values of 4 and 5 for predicting protein-containing signal peptide sequences and secreting them extracellularly.

### Transmembrane Domain Prediction

A new fasta file containing the sequences predicted by SignalP v4.1 and TargetP v1.1 was generated and submitted to the TMHMM v2.0 (Möller et al., 2001) using the website^3^. The results showing ExpAAs < 18 and the number of transmembrane structures (PredHel) was equal to “0” were selected for subsequent analysis.

### Candidate Effector Proteins Prediction

The selected sequences were extracted and submitted to the EffectorP v2.0 (Sperschneider et al., 2018a)^4^. The server is based on the molecular weight, the net charge number, and the character of contents of cysteine, serine, and tryptophan in the amino acid of the sequences. The results were displayed in text format; then, the “effector” that is the CSEP was chosen.

### Structural Characteristics Analysis of CSEPs

The number of cysteine residues and the size of the amino acids of the CSEPs were counted. Known motifs including RxLR in oomycetes, [Y/F/W]xC in powdery mildew, G[I/F/Y][A/L/S/T]R in flax rust, and [L/I]xAR, [R/K]CxxCx12H, and YxSL[R/K] in *Magnaporthe oryzae* were searched by Perl scripts. Other known rust effectors were also used to predict the homology with CSEPs. Conservative domain search was performed by Pfam. The candidate effector proteins containing no conserved structures were searched by MEME software.

### CSEPs Verification Conducted by BAX-Triggered Suppression Assays on *N. benthamiana*

The recombinant constructs with CSEPs and the PVX vector pGR107 were introduced into *A. tumefaciens* strain GV3101 by using the freeze–thaw method. GV3101 containing the corresponding recombinant constructs were cultured in LB media containing 50 mg/ml of kanamycin and 50 mg/ml rifampicin at 28°C with 220 rpm for 48 h. The bacterial pellets were harvested and washed three times with the induction buffer (10 mM MES, 10 mM MgCl_2_, 400 μM acetosyringone). To achieve an optical density of OD600 = 0.3 by buffer after resuspension, the 6-week-old leaves of *N. benthamiana* was infiltrated with a needleless syringe. To observe the suppression of cell death induced by BAX, *A. tumefaciens* cells carrying CSEPs were infiltrated initially, and BAX carrying *A. tumefaciens* cells were infiltrated at the same site after 24 h. The symptoms were monitored, and photos were taken after 5 days. *A. tumefaciens* cells carrying enhanced green fluorescent protein (eGFP) was infiltrated and used as negative control. Each assay was repeated on at least three leaves on three plants.

### Transient Expression Assay by *A. tumefaciens* on Wheat Near-Isogenic Lines

According to the method of BAX-triggered suppression assays, using the same control, the bacterial suspension with the optical density of OD600 = 1.2 was infiltrated on the fully expanded secondary leaf of wheat near-isogenic lines with a needleless syringe. The border of the infiltration area was marked with a mark pen. The inoculated leaves were photographed at 7 dpi. The assay for each gene was repeated at least twice, and each repeat consisted of 10 biological replicates.

### qRT-PCR Analyze the Expression Level of CSEPs

The total RNA of THSN-inoculated wheat leaves with *P. triticina* at 0, 6, 12, 18, 24, 36, 48, 60, 72, 96, and 120 h post-inoculation (hpi) were extracted using Plant RNA Extraction Kit (TaKaRa, Japan) following the manufacturer’s instructions. RNA was assessed by agarose gel electrophoresis and quantified by spectrophotometry. About 2.0 μg of each sample was reverse transcribed into cDNA with the 5 × All-In-One RT MasterMix (abm, Jiangsu, China). qRT-PCR reactions were run on a LightCycler detection system using SYBR Green PCR Master Mix (TransGen Biotech, Beijing, China). Elongation factor 1 (EF1) gene was used as the endogenous reference for calculating relative transcription levels. The qRT-PCR thermal profile was set as 5 min at 94°C; 40 cycles of 10 s at 94°C, 15 s at 60°C, and 20 s at 72°C; 1 cycle of 10 s at 95°C, 60 s at 65°C, and 1 s at 97°C; and a final step of 30 s at 37°C. Relative transcript levels of the test gene were calculated by the comparative threshold (2^–ΔΔCT^) method (Livak and Schmittgen, 2001). Three technical and biological replicates of each treatment were analyzed. Standard deviation was calculated in MS Excel.

## Results

### RNA-Seq Library and Comprehensive Prediction of CSEPs

We used the company platform to establish RNA-seq library. We generated about 25.81 Gb bases in total on Illumina HiSeq sequencing platform. The total numbers of Unigenes obtained from the databases for KHTT, JHKT, and THSN were 189,266 after assembly and de-redundancy using IIIumina HiSeq platform. A total of 39,741 were complete, and the longest CDS were extracted through Transdecoder software prediction for CSEPs prediction. All raw data were uploaded to National Center for Biotechnology information (NCBI) in BioProject PRJNA609405^5^.

As a result, a total of 7,158 sequences containing SP were screened out of 39,741 sequences using SignalP v4.1, D score between 0.45 and 0.97, accounting for 18% of the submitted sequences through the NN neural network and hidden Markov model method. Most of the sites of SP were located in 20–30 amino acids. Then, the sequences containing the SP were analyzed using TargetP v1.1. As a result, the locations of genes were shown as “S,” and the RC values of 1, 2, and 3 were 3,050, 1,689, and 716, respectively. In addition, there were 74 and 148 genes located in mitochondrial “M” with RC values of 4 and 5, respectively. A total of 5,677 sequences were obtained for the transmembrane domain prediction by TMHMM v2.0, and 4,514 genes were identified that met ExpAAs < 18 conditions and contained no transmembrane domain.

The 4,514 amino acid sequences were further analyzed by EffectorP v2.0, and a total of 635 sequences were obtained as “Effector,” probabilities of which ranged from 55 to 99% based on amino acid size, molecular weight, sequence length, net protein charge, the hydrophilicity, hydrophobicity, and surface tension of the proteins.

### CSEPs of *P. triticina* Have Typical Structural Characteristics

Statistical analysis of cysteine residues of 635 CSEPs revealed that only 45 effector proteins contained no cysteine and 47 genes contained one cysteine. There are 66, 50, and 80 CSEPs containing 2, 3, and 4 cysteines, respectively. The remaining 347 candidate effector proteins had more than 4 cysteine residues, of which *Pt44738* contained 28 cysteines. Amino acid quantity statistics were performed on all CSEPs, and 625 candidate effector proteins were found to be < 300 amino acids. However, 10 genes were >300 amino acids, and the largest size is 422 amino acids.

**FIGURE 1:**
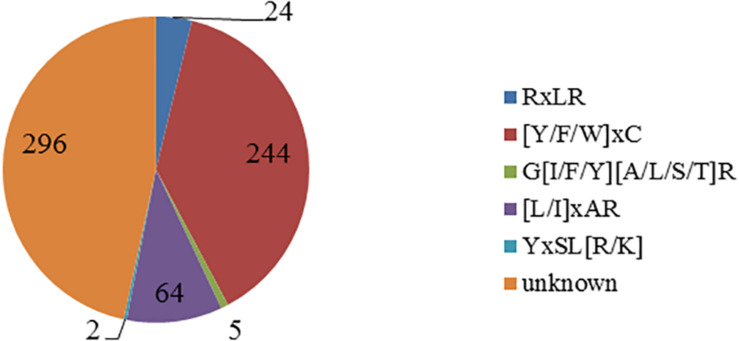
Distribution of the known motifs of 635 candidate effectors. The figure shows five known motifs and quantities.

Using the Perl script to search the known motif, a total of five motifs were found (Figure 1). There are 244 CSEPs that have the “[Y/F/W]xC” motif, the same as in powdery mildew, accounting for 38.43% of the CSEPs in our research. The numbers are 24, 5, 64, and 2 for RxLR, G[I/F/Y][A/L/S/T]R, [L/I]xAR, and YxSL[R/K] motif, respectively. However, there are unknown motifs in 296 CSEPs.

A conserved domain searching was carried out through Pfam. The results revealed that there are 16 families, 9 domains (Table 1), and 53 other types with known functions (Supplementary Figure S2).

**TABLE 1:** Types and quantities of conserved domains of the 635 candidate secreted effector proteins (CSEPs).

	**Description**	**Number**
Family	Cysteine-rich secretory protein family	8
	Thaumatin family	6
	Bowman–Birk serine protease inhibitor family	4
	Ribonuclease T2 family	6
	Ras family	2
	Aldehyde dehydrogenase family	2
	Ser–Thr-rich glycosyl-phosphatidyl-inositol-anchored membrane family	2
	Barwin family	11
	Dienelactone hydrolase family	12
	Peptidase family M48	2
	Insect pheromone-binding family, A10/OS-D	2
	ADP-ribosylation factor family	1
	2OG-Fe(II) oxygenase superfamily	3
	Glycosyl hydrolases family	15
	Papain family cysteine protease	4
	emp24/gp25L/p24 family/GOLD	1
Domain	MIR domain	2
	FAD binding domain	2
	WD domain, G-beta repeat	2
	PLAT/LH2 domain	3
	Common central domain of tyrosinase	3
	Chitin binding peritrophin-A domain	2
	Ring finger domain	2
	Leucine-rich repeat N-terminal domain	5
	CFEM domain	2

**FIGURE 2:**
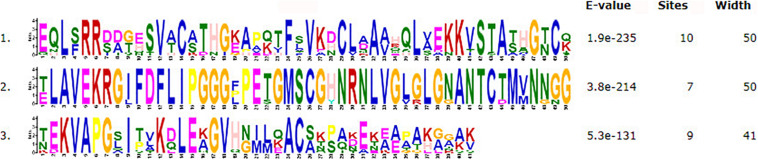
The prediction of three new motifs of candidate secreted effector proteins (CSEPs) by MEME.

**FIGURE 3:**
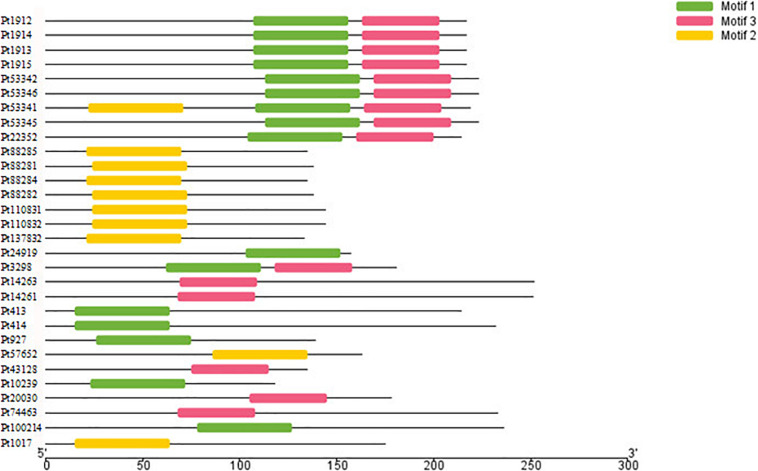
The motifs location of secreted proteins.

The motifs of 296 CSEPs with no conserved structure were searched by MEME. Three motifs with higher E value were found (Figure 2), and motifs 1–3 were present in 16, 15, and 10 CSEPs, respectively. Among them, nine CSEPs contained both motifs 1 and 2. Moreover, *Pt53341* contained three motifs (Figure 3).

### Correlation Analysis Between *P. triticina* CSEPs and Other Rust Sequences

The 635 *P. triticina* CSEPs were compared with previously reported CSEPs of *P. triticina*, *P. striiformis* f. sp. *tritici*, and *P. gramminis* f. sp. *tritici* (Cantu et al., 2013) using TBtools v0.66839 (Chen et al., 2018). We found that 339 sequences share identity with genes in *P. triticina*. A total of 204 CSEPs have 100% identity with the sequences in BBBD (*P. triticina*), and the similarities of the remaining CSEPs with BBBD (*P. triticina*) were more than 70%. Ninety CSEPs were more than 70% similar to that in *P. gramminis* f. sp. *tritici*. The identity between *Pt20856* and PGTG_024280 was 94%. Fifty-four CSEPs shared sequence similarity with *P. striiformis* f. sp. *tritici*, and the identity of *Pt72634* with *PST43_01110* was 90%. We also found that 20 genes of 635 CSEPs were able to align simultaneously to known effector proteins in *P. triticina*, *P. striiformis* f. sp. *tritici*, and *P. gramminis* f. sp. *tritici* with 70% identity (Table 2).

**TABLE 2:** Identity of the 20 candidate effector proteins to other wheat rust fungi.

**Pt CSEPs ID**	**Blast2GO vs. (PGT and PST) CSEPs (aa)**					
	**PT CSEPs ID**	**Identity**	**PGT CSEPs ID**	**Identity**	**PST CSEPs ID**	**Identity**
Pt396	PTTG_07398	100%	PGTG_183090	93%	PST21_17770	80%
Pt85431	PTTG_00127	99%	PGTG_059190	92%	PST877_02314	86%
Pt85432	PTTG_00127	99%	PGTG_059190	91%	PST877_02314	86%
Pt14249	PTTG_08828	100%	PGTG_100450	89%	PST43_16562	76%
Pt15546	PTTG_00528	100%	PGTG_156230	86%	PST130_13123	78%
Pt22131	PTTG_09156	100%	PGTG_008980	86%	PST0821_05872	82%
Pt77192	PTTG_06434	100%	PGTG_107890	86%	PST21_19826	64%
Pt225	PTTG_04779	100%	PGTG_183090	85%	PST21_17770	85%
Pt23713	PTTG_25228	100%	PGTG_113480	84%	PST21_18447	84%
Pt11621	PTTG_25160	99%	PGTG_134580	84%	PST21_11336	79%
Pt89751	PTTG_09007	100%	PGTG_044810	83%	PST43_08676	72%
Pt11623	PTTG_25160	98%	PGTG_134580	83%	PST21_11336	79%
Pt15547	PTTG_10178	100%	PGTG_004950	81%	PST0821_01464	82%
Pt11730	PTTG_01597	100%	PGTG_187770	81%	PST0821_06071	82%
Pt8638	PTTG_01578	100%	PGTG_198270	81%	PST21_16702	77%
Pt28907	PTTG_02997	100%	PGTG_049500	80%	PST43_03428	72%
Pt23611	PTTG_08287	100%	PGTG_203880	79%	PST877_20525	79%
Pt38452	PTTG_01597	98%	PGTG_187770	79%	PST0821_06071	80%
Pt13631	PTTG_05347	100%	PGTG_128320	75%	PST21_19108	72%
Pt17178	PTTG_01156	100%	PGTG_082680	74%	PST877_15546	73%

### Tested CSEPs Successfully Suppressed PCD Induced by BAX on *N. benthamiana*

**FIGURE 4:**
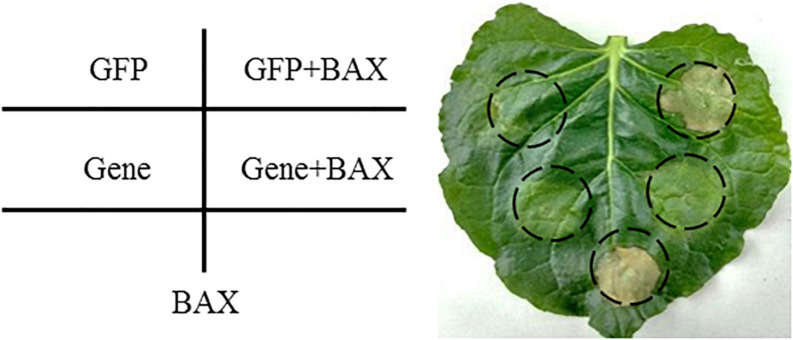
Transient expression of candidate secreted effector proteins (CSEPs) in *N. benthamiana* suppressed programmed cell death triggered by BAX schematic. *N. benthamiana* leaves were infiltrated with *A. tumefaciens* cells containing PVX vector carrying enhanced green fluorescent protein (eGFP) (negative control) or CSEPs, respectively, followed by inoculating with *A. tumefaciens cells* carrying PVX:BAX after 24 h. Photos were taken 5 days after infiltration.

We determined the function of randomly selected 30 CSEPs (Table 3) using an *A. tumefaciens*-mediated transient expression assay in *N. benthamiana* (Figure 4). The phenotypes were observed 5 days post-BAX infiltration. The infiltration of the control bacteria suspension carrying pGR107-eGFP did not result in PCD, and the sites infiltrated with pGR107-eGFP and pGR107-BAX exhibited obvious cell death on *N. benthamiana*. However, the infiltration with pGR107-CSEPs successfully suppressed PCD induced by BAX (Supplementary Figure S3).

**TABLE 3:** Sequence characteristics of the 30 candidate effectors.

**Gene**	**Size (aa)**	**Cys**	**Best hit in NCBI database**	**E-value**	**Domain**	**Known motif**
Pt7277	241	1	Hypothetical protein PTTG_12701	5.E–179	NO	NO
Pt10853	129	4	Hypothetical protein PTTG_11646	6.E–67	NO	NO
Pt12858	186	5	Copper/zinc superoxide dismutase [BBBD]	1.E–136	NO	NO
Pt16481	147	0	Hypothetical protein PTTG_04158	1.E–90	NO	NO
Pt5794	133	5	Hypothetical protein PTTG_12430	1.E–95	NO	FxC
Pt494	115	3	Hypothetical protein PTTG_12223	2.E–50	NO	NO
Pt3081	197	4	Hypothetical protein PTTG_05750	1.E–142	NO	NO
Pt97002	124	10	Hypothetical protein PTTG_25643	2.E–64	NO	F/YxC
Pt15387	219	5	Hypothetical protein PTTG_05844	1.E–133	NO	NO
Pt34354	218	14	Hypothetical protein PTTG_26041	7.E–153	NO	YxC
Pt88286	135	4	Hypothetical protein PTTG_04312	3.E–76	NO	NO
Pt15525	229	6	Hypothetical protein PTTG_00114	2.E–157	NO	NO
Pt8502	266	3	Hypothetical protein PTTG_01715	2.E–73	NO	NO
Pt15546	245	6	Hypothetical protein PTTG_00528	0. E + 00	NO	NO
Pt20779	125	10	Hypothetical protein PTTG_05518	3.E–87	NO	FxC
Pt8638	201	6	Hypothetical protein PTTG_01578	9.E–136	NO	NO
Pt77192	269	8	Hypothetical protein PTTG_06434	0. E + 00	NO	NO
Pt1625	242	4	Hypothetical protein PTTG_12483	6.E–151	NO	FxC
Pt23713	144	5	Hypothetical protein PTTG_25228	8.E–104	NO	FxC
Pt36553	246	6	Hypothetical protein PTTG_03168	2.E–153	NO	NO
Pt18222	194	6	Hypothetical protein PTTG_25283	3.E–121	NO	NO
Pt36853	192	9	Hypothetical protein PTTG_11686	1.E–142	NO	YxC
Pt2567	168	1	Hypothetical protein PTTG_28625	4.E–118	NO	NO
Pt94682	217	6	Hypothetical protein PTTG_05290	3.E–147	NO	NO
Pt29088	248	1	Hypothetical protein PTTG_02768	2.E–167	NO	NO
Pt16552	201	6	Hypothetical protein PTTG_01578	9.E–136	NO	NO
Pt16	127	5	Hypothetical protein PTTG_02728	7.E–23	NO	NO
Pt17	113	5	Hypothetical protein PTTG_26540	2.E–34	NO	WxC
Pt3372	139	0	Hypothetical protein PTTG_25108	7.E–86	NO	NO
Pt14306	135	7	Hypothetical protein PTTG_12514	2.E–82	NO	NO

### HR Can Be Specifically Induced by CSEPs of *P. triticina* on Wheat Near-Isogenic Lines

**FIGURE 5:**
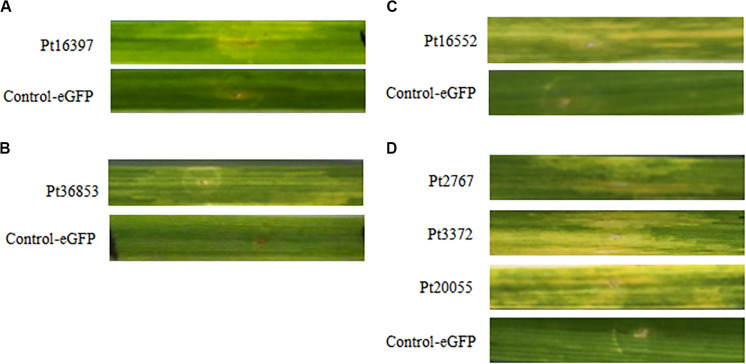
Six candidate secreted effector proteins (CSEPs) of *P. triticina* can especially induce hypersensitive response (HR) on the wheat near-isogenic lines. **(A–C)** Phenotype of *Pt16397*, *Pt36853*, and *Pt16552* inoculated on wheat near-isogenic lines TcLr26, TcLr47, and TcLr41, respectively. **(D)** *Pt7277*, *Pt88286*, and *Pt3372* play role in TcLr42.

According to the results of BAX assays, six CSEPs were chosen to verify the function using the transient expression infiltrated by *A. tumefaciens* method on wheat near-isogenic lines. The results showed that eGFP control cannot induce HR in any wheat near-isogenic lines, but chlorotic phenomenon was observed on wheat near-isogenic line leaves after infiltrated with CSEPs, including *Pt7277*, *Pt88286*, *Pt3372* in TcLr42; *Pt77192* in TcLr26; *Pt36853* in TcLr47; and *Pt16552* in TcLr41 (Figure 5). The results suggested that the overexpression of CSEPs of *P. triticina* can effectively induce HR.

### The Expression Pattern of CSEPs of *P. triticina* at Different Development Stages

**FIGURE 6:**
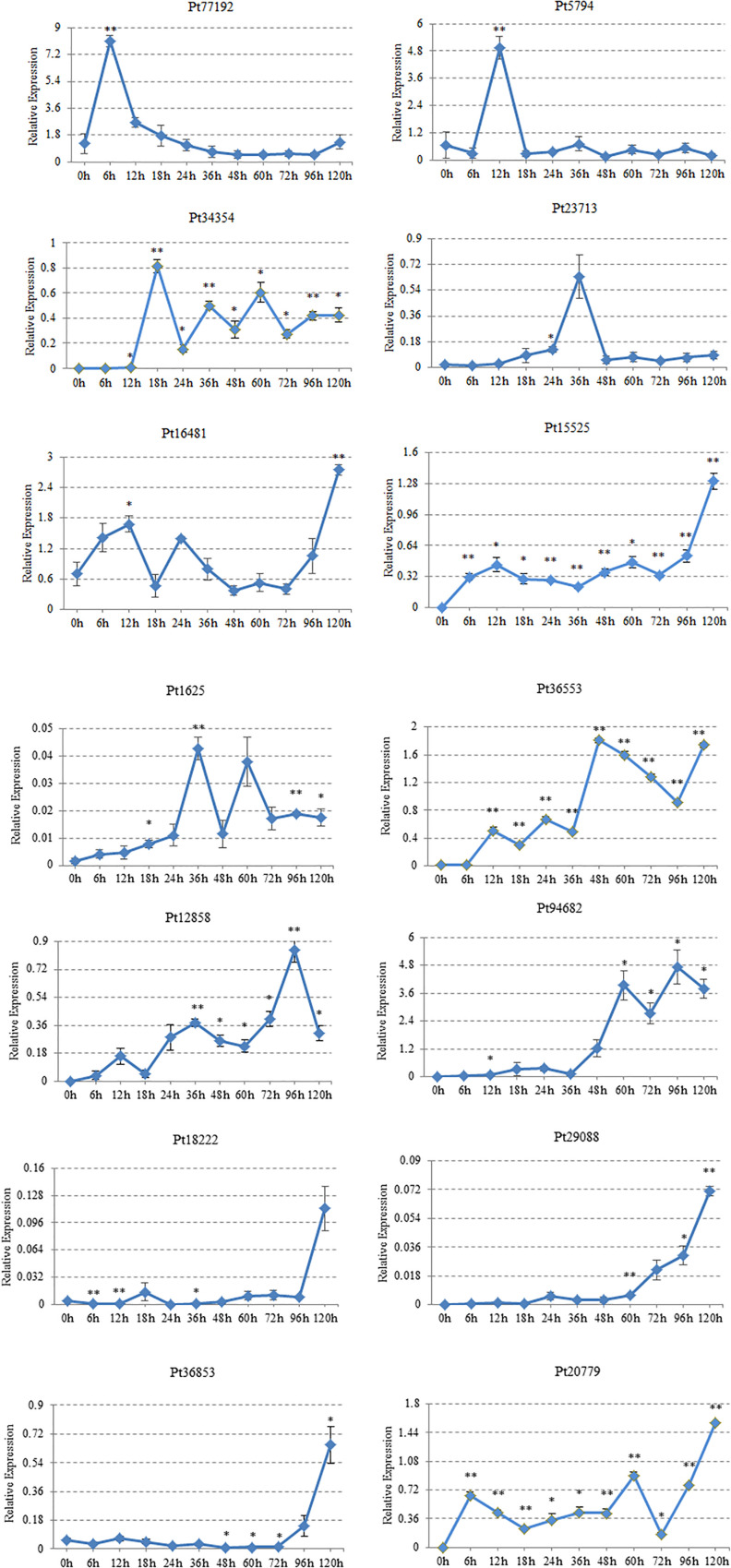
Gene expression profiles for 14 candidate effector proteins. Plant samples were taken at these inoculation time points (X-axis). h, hours post-inoculation (hpi). Gene expression level, relative expression (Y-axis). The expression levels of each gene were calculated according to the 2^–ΔΔCT^ method compared with an endogenous gene EF1. Three technical replicates and biological repetition for each treatment were analyzed. Calculations of the mean and standard error were performed using Microsoft Excel software. Asterisks indicate significant differences (^∗∗^*P* < 0.01, ^∗^*P* < 0.05) relative to the US sample determined using Student’s *t*-test.

The expression characteristics of 14 CSEPs on “Thatcher” were analyzed by qRT-PCR. We found that *Pt77192* was upregulated during 0–6 hpi and reached the peak at 6 hpi; then, it showed a significant downward trend when interacted with “Thatcher.” *Pt5974* peaked at 12 hpi, and the expression was downregulated during 18–120 hpi. *Pt34354* showed the highest expression at 18 hpi, then decreased, and increased at 36 and 60 hpi, respectively. Both *Pt23713* and *Pt1625* reached the expression peak at 36 hpi. *Pt1625* decreased rapidly at 48 hpi after upregulating at 36 hpi and reached an expression peak at 60 hpi, followed by downregulation. *Pt36553* was not expressed during 0–6 hpi, but it was expressed in wavy trend during 12–36 hpi, peaked at 48 hpi, then was downregulated at 96 hpi. *Pt12858* was upregulated at 12 and 36 hpi and peaked at 96 hpi. *Pt96482* showed almost no expression during 0–36 hpi but began to climb two peaks at 60 and 96 hpi, respectively. *Pt18222*, *Pt29088*, and *Pt36853* reached the peak expression at 120 hpi. *Pt18222* showed a low expression level at 0–96 hpi and peaked at 120 hpi. *Pt29088* showed a low expression level during 0–60 hpi and gradually upregulated at 72 hpi until reaching the expression peak at 120 hpi. *Pt36853* started to express at 72 hpi, and the highest expression level was at 120 hpi. The expressions of *Pt20779*, *Pt16481*, and *Pt15525* showed the same trend. They all showed a wavy trend during 0–72 hpi, upregulated at 72 hpi, and then peaked at 120 hpi (Figure 6).

## Discussion

### Utilizing Multiple Approaches Can Improve the Prediction Efficiency and Accuracy of CSEPs

Effector proteins are important virulence or avirulence factors in pathogens. Some fungal CSEPs have certain characteristics, such as containing conservative motifs RxLR in oomycetes and [Y/F/W]xC in powdery mildew or have sequence similarity. However, most fungal effectors generally do not exhibit a high degree of amino acid sequence conservation. Therefore, the prediction of effector mainly depends on the method of bioinformatics. Previous study has verified that this method is useful to predict the secreted proteins of various pathogens (Sonah et al., 2016). The first step of the prediction of CSEPs is usually to predict extracellular secretion signals. SignalP v4.1 based on SP site combines with several artificial neural networks for predicting SP/non-SP. D-score values can be set (to distinguish between SP and non-SP values) as well as the minimum position in the sequence. The use of SignalP v4.1 makes the screening conditions more stringent and more accurate and greatly narrows the scope of secreted proteins.

Some CSEPs have plant localization signals; different effectors may localize to plant cell membranes, cytoplasm, endoplasmic reticulum, Golgi, mitochondria, chloroplast, and nucleus. TargetP can predict protein localization in large quantities, which provides valuable clues for determining the site of action of plant intracellular effector. The accuracy rate for plants and non-plants are up to 85 and 90%, respectively (Sonnhammer et al., 1998; Emanuelsson et al., 2000).

The presence of signal peptides and plant localization signals is not sufficient to indicate that a protein is secreted. SP can also direct proteins to cytoplasmic organelles. Therefore, distinguishing SP and TM is very important (Robinson et al., 2012; Reithinger et al., 2013). There are several predictive servers available for predicting TM regions. TMHMM is a tool for predicting transmembrane domains of proteins using hidden Markov Models. It covers the prediction of structural and chemical properties in the transmembrane region as well as inside and outside the membrane and predicts the charge, hydrophobicity, membrane protein topology, and helical length of the transmembrane region. The software can effectively distinguish SP and TM to improve prediction accuracy.

A number of effector proteins can be predicted using the above three software. A total of 4,514 candidate effectors were predicted based on SignalP v4.1, TargetP v1.1, and TMHMM v2.0. Based on the properties of the effector proteins, we further predicted them using EffectorP. EffectorP v1.0, an effector protein prediction software, was developed based on a series of properties of proteins. It is the first prediction program based on machine training to predict fungal effectors (Sperschneider et al., 2016). This program promotes functional studies of fungal effector and enhances our understanding of plant–pathogen interactions. However, EffectorP v1.0 uses a small range of screening criteria, including amino acid frequency, molecular weight, sequence length, and net protein charge. The software was optimized with adding filters based on the hydrophilicity, hydrophobicity, and surface tension of the proteins and developed into EffectorP v2.0 in 2018 (Sperschneider et al., 2018a). For the accuracy of the prediction of two versions, corresponding experiments were conducted. CSEPs of 13 fungi, such as *Colletotrichum higginsianum*, *Cladosporium fulvum*, *M. oryzae*, *Blumeria graminis* f. sp. *hordei*, *Melampsora larici-populina*, *P. gramminis* f. sp. *tritici*, *Ustilago maydis*, and *Leptosphaeria maculans* in the infection stage, were predicted (Sperschneider et al., 2018a). They found that 12 species were predicted by EffectorP v2.0, and the prediction rate reached to 92.3%. However, 10 species were predicted by EffectorP v1.0, and the forecast rate is 76.9%. At the same time, researchers combined the two versions to predict 115 effectors and found that the accuracy is as high as 90.5%, which is higher than the probability of using only EffectorP v1.0 or v2.0. The combination of the two versions is the most powerful means to predict effector, but numerous effector proteins are lost. Thus, the ability of EffectorP v2.0 to predict fungal effector proteins is higher than that of EffectorP v1.0, and the range of prediction is also beyond the combined two versions. Therefore, we chose EffectorP v2.0 for the effector protein prediction on the base of screening by SignalP v4.1, TargetP v1.1, and TMHMM v2.0. Finally, a total of 635 CSEPs were obtained. EffectorP v2.0 is a powerful tool for predicting fungal candidate secreted effector proteins, but more experiments are needed to support the prediction results, such as host-induced gene silencing (HIGS) and transgenosis, to discover the function of the effector. Moreover, yeast two-hybrid system (Y2H), bimolecular fluorescence complementation (BiFC), and immunoprecipitation are essential to identify the interaction target with host.

### CSEPs of *P. triticina* Contained Some Known Motifs of Other Pathogens

Previous studies has confirmed that the effector proteins contained the motifs, such as RxLR in oomycetes, [L/I]xAR and YxSL[R/K] in rice blast fungus, [Y/F/W]xC in powdery mildew, and G[I/F/Y][A/L/S/T]R in flax rust. The consensus sequence RxLR motif was originally identified by comparing effectors from *Hyaloperonospora arabidopsidis*, *Phytophthora infestans*, and *Phytophthora sojae* but found no RxLR motif in fungal effectors (Rehmany et al., 2005). Interestingly, in this study, 24 CSEPs with RxLR motif were found from 635 CSEPs of *P. triticina*. Moreover, we found 244 candidate effector proteins with [Y/F/W]xC motif in powdery mildew, which is larger than 57 found in wheat leaf rust fungi by Godfrey et al. (2010). The results demonstrated that the effector proteins produced by *P. triticina* have similar structure as the *B. graminis* f. sp. *hordei* effectors. The CSEPs with [Y/F/W]xC motif accounted for 38.43% of all the CSEPs in our library, suggesting that the motif plays fundamental roles in haustoria function. Furthermore, five effector proteins contain G[I/F/Y][A/L/S/T]R motif, which was reported in flax rust. Two YxSL[R/K] motifs and 64 [L/I]xAR motifs in *M. oryzae* were found in *P. triticina* (Figure 1). No known motifs were found in the other 296 CSEPs, representing 46.6% of all the CSEPs (Figure 1), indicating that the CSEPs of *P. triticina* have certain sequence and hereditary homology with the known fungal effector proteins. This also indicated that the CSEPs of *P. triticina* predicted by EffectorP v2.0 were accurate.

### CSEPs of *P. triticina* Contained New Motifs and Homology to *P. gramminis* f. sp. *tritici* and *P. striiformis* f. sp. *tritici*

Three kinds of motifs that differed from other fungi were first discovered by MEME prediction for 296 CSEPs that have no known motifs (Figure 3), indicating that the CSEPs in *P. triticina* are diverse and specific. However, the function of the known motif remains to be confirmed experimentally.

To demonstrate the accuracy of the results, we also blast the 635 CSEPs with secreted proteins of *P. triticina*, *P. gramminis* f. sp. *tritici*, and *P. striiformis* f. sp. *tritici* reported by Cantu et al. (2013) using TBtools v0.66839 (Chen et al., 2018). The results showed that 339 genes were identical to genes in BBBD isolate of *P. triticina.* In addition, 90 and 54 candidate effector proteins were identical to sequences in *P. gramminis* f. sp. *tritici* and *P. striiformis* f. sp. *tritici*, respectively, and the identities ranged from 70 to 90%. The results indicated that the CSEPs of *P. triticina* were related to that in other rust fungi, and they were more specific and difficult to obtain by homologous sequence method.

Structural features of fungal effector proteins include extracellular localization, small sequence, or low molecular weight (e.g., sequences are < 300 AA or sequences are <30 kDa) and enriched in cysteine (e.g., sequences contain more than or equal to four cysteine). Figueroa et al. (2015) found that effector ToxB had 87 amino acids and 4 cysteines in *Pyrenophora tritici-repentis*. However, previous studies found that there are some exceptions in the CSEPs. For example, the *P. sojae* effector protein PsXEG1 contains only two cysteines (Ma et al., 2015). The flax rust effector protein *AvrM* has 314 amino acids but only 1 cysteine (Catanzariti et al., 2006). In this study, we predicted 635 CSEPs, and most of them were conventional. The number of cysteine residues in 427 genes were ≥4, especially *Pt44738* that contained 28 cysteines. The remaining 208 genes contained less than four cysteines. The molecular weight of 625 out of 635 candidate effector proteins were < 300 amino acids. However, the size of 10 genes were >300 amino acids. For example, the molecular weight of *Pt91311* were 422 amino acids, indicating that the CSEPs of *P. triticina* has abundant specificity. Therefore, screening in a broad condition may filter out some effectors. More experimental data are needed to optimize the screening method, such as using the known effector information, expanding screening criteria, etc.

### CSEPs of *P. triticina* May Confer Many Functions

At present, some CSEPs in the plant phytopathology have classified biological functions. Based on the Pfam domain search, we found some related families and domains in this study. Among them, 27 genes were found to belong to the hydrolase family, including the glycosyl hydrolase family and the dienelactone hydrolase family. There were 10 CSEPs assigned to the known proteins with hydrolase (Cuomo et al., 2017). Previous studies suggested that hydrolases such as glucanase and chitinase were involved in the fungal spore germination process (Mouyna et al., 2013), hypha growth and branching emergence (Wessels, 1993; Adams et al., 2004; Sandini et al., 2007), hyphae fusion (Glass et al., 2000), basidiomycete fruiting development (Kamada and Takemaru, 1977; Sakamoto et al., 2005; Tao et al., 2013), and the autolysis acting in releasing the fruiting bodies of the spores (Kües, 2000; Liu et al., 2015). These are all related to the growth and development of fungi.

Another large group found in six CSEPs belonged to the Thaumatin family. Grenier et al. (2000) first reported this family in basidiomycetes. The gene encoded by the Thaumatin-like protein (TLP) was found in *Cryptococcus neoformans* and participated in the degradation of the plant lentinan and the cell wall during the microspore diffusion process (Sakamoto et al., 2006). Among the 1,358 wheat leaf rust CSEPs (Cuomo et al., 2017), six genes contain thaumatin domain, but their functions have not been reported. In our research, 11 genes belonged to the Barwin family (Table 1) known elicitor cerato-platanin of fungi (Pazzagli et al., 1999), which are more than that found by Bruce et al. (2014), who found only one CSEP that was associated with Barwin through performing transcriptome analysis on 6 *P. triticina* races and obtained 532 CSEPs. It has also been reported that proteins containing this domain promoted the successful colonization of *C. neoformans* in the lungs of mammals (Camacho et al., 2019). In addition, these 635 candidate effector proteins included other family domains (Table 1), such as the ribonuclease family, the peptidase family, the aldehyde dehydrogenase family, the Bowman–Birk serine protease inhibitor family, the Ras family, etc. However, the functions of these families in fungi need to be further explored.

We also found 9 domains in the 635 CSEPs (Table 1), of which the CFEM domain is an extracellular plasma membrane protein containing eight cysteine residues, a unique protein associated with fungal pathogenesis. There are other enzymes and proteins (Supplementary Figure S2), such as copper/zinc superoxide dismutase, polysaccharide deacetylase, hydrophobic protein, phytosulfokine precursor protein, etc. They may play a role in the interaction between pathogens and hosts, but the functions of these conserved domains need to be further studied.

### Heterologous Transient Expression Verified the CSEPs of *P. triticina*

*P. triticina* is an obligate biotrophic fungus, and it lacks an effective genetic transformation system to be verified *in vitro*. Therefore, *Agrobacterium*-mediated transient transformation system was applied in assessing the function of effector. Researches had shown that cell necrosis induced by BAX in plants was similar to the HR induced by pathogen in physiological characteristics (Lacomme and Santa Cruz, 1999). It is speculated that the inhibition of BAX-induced PCD reflected the virulent function of inhibiting host defense response of CSEPs. It is a simple and effective way to identify the function of effector. Our experiment utilized heterologous expression system and the mouse-apoptotic gene BAX elicitor to detect the function of the 30 CSEPs of *P. triticina* in *N. benthamiana* 5 days after inoculation with *A. tumefaciens*. The results showed that 30 CSEPs of *P. triticina* that we tested inhibited BAX-induced PCD, and the inhibition ratio was up to 100%; this hints that the screened CSEPs of *P. triticina* are more accurate.

### Instantaneous Expression of the CSEPs on Wheat Near-Isogenic Lines

We conducted cell-death suppression assay for selected 30 CSEPs using BAX elicitor *in vitro*. However, these results are still indirect evidence. Therefore, we used *A. tumefaciens*-mediated instantaneous expression to directly test wheat near-isogenic lines. The results showed that six CSEPs of *P. triticina* can especially induce HR on different wheat cultivars. *Pt77192*, *Pt36853*, and *Pt16552* acted in TcLr26, TcLr47, and TcLr41, respectively. Interestingly, *Pt7277*, *Pt88286*, and *Pt3372* played role in TcLr42 at the same time. The reason why different effectors from the same *P. triticina* could stimulate the same R protein is that effector redundancy is a product of arms race coevolution between pathogens and hosts; they may be recognized by the R protein *Lr42* in different ways, such as the guard mode or decoy mode. In biology, genetic redundancy contributes to robustness in the face of a changing environment. Similarly, plant–pathogen populations benefit from carrying a redundant reservoir of effectors to counteract host immunity (Win et al., 2012). This could also explain why different effectors can target the same host protein whether the effectors are related.

### qRT-PCR Showed the Expression Characteristics of CSEPs of Wheat Leaf Rust

The production and expression of CSEPs differed at different stages. Real-time quantitative expression analysis can reveal the expression characteristics of CSEPs, which help to prove that CSEPs are effectors indirectly through the expression time points. In this study, there were 14 genes in the stage of *P. triticina* infecting on wheat analyzed by qRT-PCR. According to the results of transcriptome sequencing, there were 14 genes upregulated in the germination stage and the infection stage of *P. triticina* race JHKT. From the results of qRT-PCR, the expression levels of *Pt18222*, *Pt29088*, and *Pt36853* reached the peak at 120 hpi, while *Pt12858* and *Pt96482* showed the highest expression at 96 hpi. *Pt20779*, *Pt16481*, and *Pt15525* were expressed in the whole stage of interaction between leaf rust and host. *Pt77192*, *Pt5974*, *Pt34354*, *Pt23713*, *Pt1625*, and *Pt36553* were expressed at 6, 12, 18, 36, and 48 hpi. These results indicated that these genes were induced when interacting with the host and were upregulated in the different interaction stages. Therefore, they may act as candidate effector proteins and may play different roles in the pathogenic process.

### The Future of Prediction of Fungal Effector Proteins

In this research, we chose the CDS in the cDNA library obtained by RNA-seq sequencing to screen the effectors; this may not be a full-length sequence due to splicing, de-redundancy, etc. Therefore, the results predicted by software alone are not all of the CSEPs of pathogens. It is necessary to fully understand CSEPs with a large number of related studies of different pathogenic strains. Although we use the multiple prediction approaches to predict the effectors in *P. triticina* during the different interaction stages with wheat cultivar “Thatcher,” structural analysis, heterologous expression, and other methods are used to improve the efficiency and accuracy of the CSEPs screening of wheat leaf rust. Some CSEPs were lost due to the existence of specificity of candidate secreted effector proteins. For example, researchers found that some effector proteins have no signal peptide (Prasad et al., 2019). Additionally, the tools that we used were based on the characters of the cloned effector proteins, so further functional verification is required. It is believed that along with the deepening of research and the improvement of methods, the CSEPs will be enriched and improved.

## Conclusion

Integrated software including SignalP v4.1, TargetP v1.1, TMHMM v2.0, and EffectorP v2.0 for analyzing the RNA-seq data are efficient in predicting CSEPs, and 635 CSEPs were screened from RNA-seq data of 3 *P. triticina* strains – KHTT, JHKT, and THSN – at three different stages in this way. The CSEPs of *P. triticina* contain various family domains including glycosyl hydrolase family, Thaumatin family, Barwin family, etc. Several known motifs such as RxLR, [Y/F/W]xC, G[I/F/Y] [A/L/S/T]R, [L/I]xAR, and YxSL[R/K] and three *de novo* motifs were identified. CSEPs found in *P. triticina* have above 70% identity to the sequences in *P. gramminis* f. sp. *tritici* and *P. striiformis* f. sp. *tritici*. The tested CESPs can inhibited BAX-induced PCD and the inhibition ratio up to 100%. The results laid a solid foundation for understanding the interaction mechanism between *P. triticina* and wheat, enriching the pathogenic mechanism of obligate biotrophic fungal and utilizing avirulent genes.

## Data Availability Statement

The datasets presented in this study can be found in online repositories. The data has deposited in the NCBI. The SRA accession numbers are SRR11929934, SRR11929933, SRR11929922, SRR11929915, SRR11929914, SRR11929913, SRR11929912, SRR11929911, SRR11929910, SRR11929909, SRR11929932, SRR11929931, SRR11929930, SRR11929929, SRR11929928, SRR11929927, SRR11929926, SRR11929925, SRR11929924, SRR11929923, SRR11929921, SRR11929920, SRR11929919, SRR11929918, SRR11929917, and SRR11929916. The TSA accession number is GISY00000000 and consists of sequences and consists of sequences GISY01000001–GISY01092094.

## Author Contributions

YZ, WY, and DL contributed to the design of the work. WY supervised the experiments. JW constructed RNA-Seq library. YZ analyzed the sequencing data. YZ and NZ submitted the data to NCBI. YZ, YQ, and JL performed the experiments. YZ wrote the manuscript. JW, RA, NZ, and WY revised the manuscript.

## Conflict of Interest

The authors declare that the research was conducted in the absence of any commercial or financial relationships that could be construed as a potential conflict of interest.

## Footnotes

1 http://www.cbs.dtu.dk/services/SignalP/

2 http://www.cbs.dtu.dk/services/TargetP/

3 http://www.cbs.dtu.dk/services/TMHMM/

4 http://effectorp.csiro.au/

5 https://dataview.ncbi.nlm.nih.gov/object/PRJNA609405?reviewer=j2png1bo7u3jlpp6h3amhk0jfm

## Supplementary Material

FIGURE S1 The flow chart of the prediction of candidate secreted effector proteins.

FIGURE S2 Domains of other known functions of 635 candidate effector proteins.

FIGURE S3 Thirty candidate effector proteins that can inhibit the cell death triggered by BAX. 1–30 represents the candidate effector proteins *Pt7277*, *Pt10853*, *Pt12858*, *Pt16481*, *Pt5794*, *Pt494*, *Pt3081*, *Pt97002*, *Pt15387*, *Pt34354*, *Pt88286*, *Pt15525*, *Pt8502*, *Pt15546*, *Pt20779*, *Pt8638*, *Pt77192*, *Pt1625*, *Pt23713*, *Pt36553*, *Pt18222*, *Pt36853*, *Pt2567*, *Pt94682*, *Pt29088*, *Pt16552*, *Pt16*, *Pt17*, *Pt3372*, *Pt14306*.

TABLE S1 Primers used in the HR suppression assays.

TABLE S2 Primers used in the qRT-PCR assays.
