# Supplementary figures and images for: Predicating the Effector Proteins Secreted by Puccinia triticina Through Transcriptomic Analysis and Multiple Prediction Approaches
Source: Front Microbiol. 2020 Sep 22;11:538032. doi: 10.3389/fmicb.2020.538032 (PMC7536266; doi:10.3389/fmicb.2020.538032)

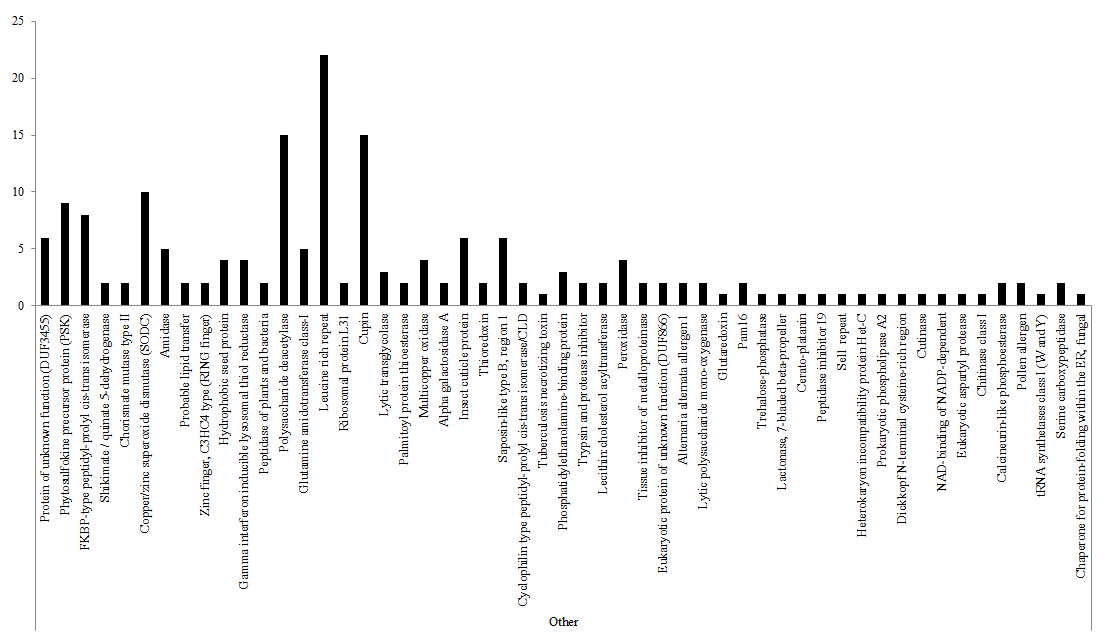

Supplement: FIGURE S1 — The flow chart of the prediction of candidate secreted effector proteins. [file Image_1.TIF]

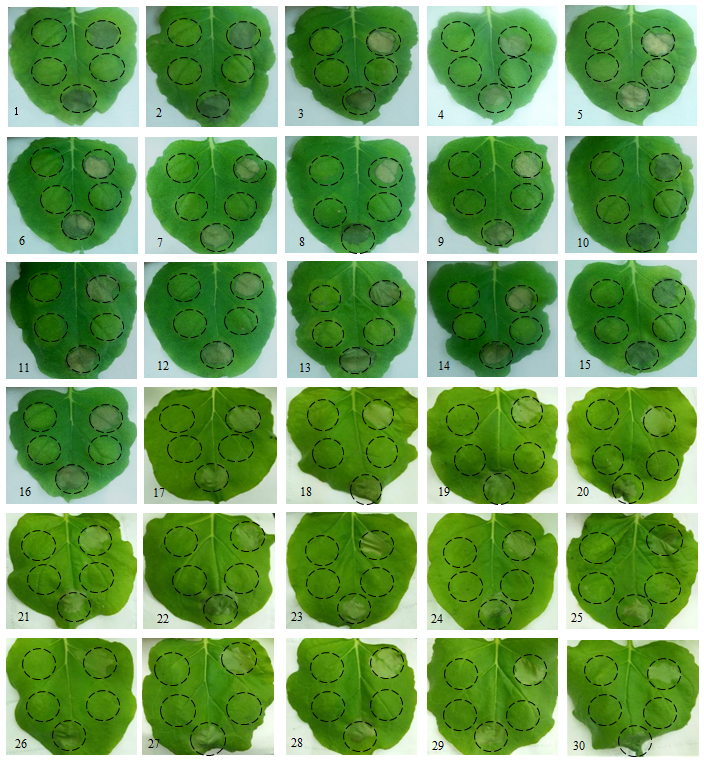

Supplement: FIGURE S2 — Domains of other known functions of 635 candidate effector proteins. [file Image_2.TIF]

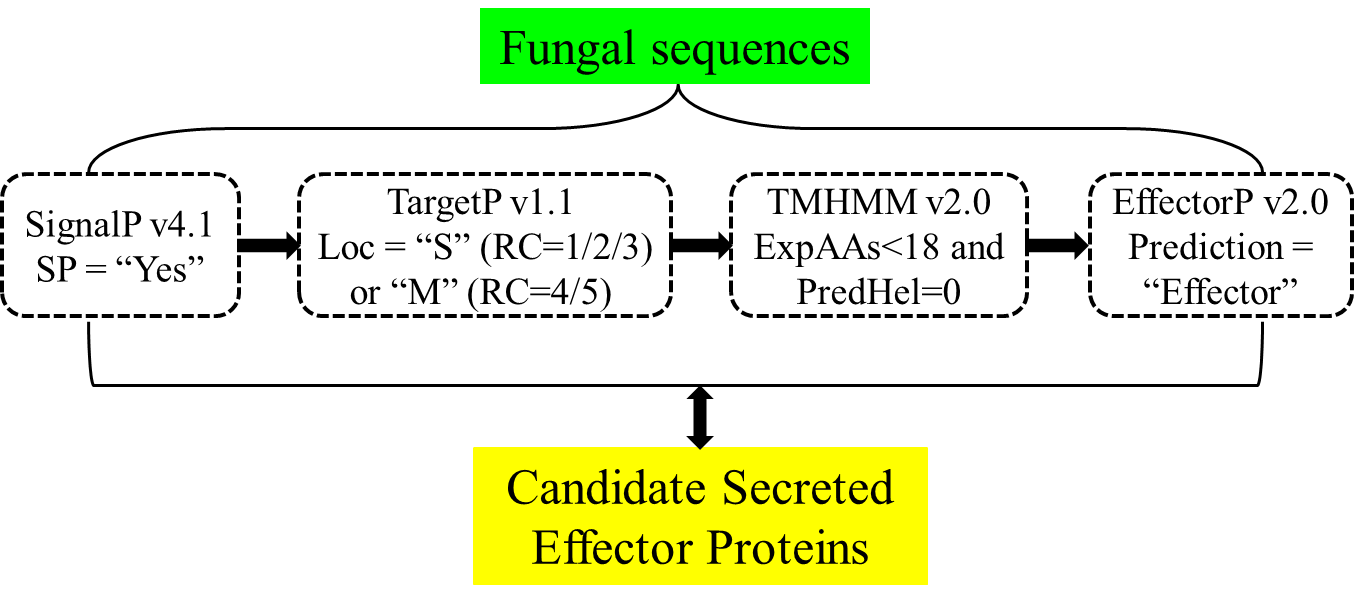

Supplement: FIGURE S3 — Thirty candidate effector proteins that can inhibit the cell death triggered by BAX. 1–30 represents the candidate effector proteins Pt7277, Pt10853, Pt12858, Pt16481, Pt5794, Pt494, Pt3081, Pt97002, Pt15387, Pt34354, Pt88286, Pt15525, Pt8502, Pt15546, Pt20779, Pt8638, Pt77192, Pt1625, Pt23713, Pt36553, Pt18222, Pt36853, Pt2567, Pt94682, Pt29088, Pt16552, Pt16, Pt17, Pt3372, Pt14306. [file Image_3.TIF]
