# Supplementary material for: Predicating the Effector Proteins Secreted by Puccinia triticina Through Transcriptomic Analysis and Multiple Prediction Approaches
Source: Front Microbiol. 2020 Sep 22;11:538032. doi: 10.3389/fmicb.2020.538032 (PMC7536266; doi:10.3389/fmicb.2020.538032)
Supplement: TABLE S1 — Primers used in the HR suppression assays. [file Data_Sheet_1.docx]

**Supplementary Table S1.** Primers used in the experiment for BAX- triggered suppression assays.

| Primer name | Primer 5'-3' | Purpose |
| --- | --- | --- |
| BAX-Pt7277-F | CCCATCGATATGATGATTACAGATCCGGT | Clone CSEPs to PVX for expression in *N. benthamiana* |
| BAX-Pt7277-R | TCCCCCGGGGGCAACGACTACCGGGTGC | Clone CSEPs to PVX for expression in *N. benthamiana* |
| BAX-Pt10853-F | CCCATCGATACTCATCCTAAAGGATGTTT | Clone CSEPs to PVX for expression in *N. benthamiana* |
| BAX-Pt10853-R | TCCCCCGGGAGCGAGGGCAAGAAAAATC | Clone CSEPs to PVX for expression in *N. benthamiana* |
| BAX-Pt12858-F | CCCATCGATCTGTCGGTCAAACCACCCTA | Clone CSEPs to PVX for expression in *N. benthamiana* |
| BAX-Pt12858-R | TCCCCCGGGGACCCCTGGCGCAAAGGCC | Clone CSEPs to PVX for expression in *N. benthamiana* |
| BAX-Pt16481-F | CCCATCGATAACTCGATCGTATCGCCGACG | Clone CSEPs to PVX for expression in *N. benthamiana* |
| BAX-Pt16481-R | TCCCCCGGGGTTCGGCTTGATCGTCACAG | Clone CSEPs to PVX for expression in *N. benthamiana* |
| BAX-Pt5794-F | CCCATCGATGATGTATTCGTGTGCCCCACG | Clone CSEPs to PVX for expression in *N. benthamiana* |
| BAX-Pt5794-R | TCCCCCGGGTACATGGGTGGTCAGTATATT | Clone CSEPs to PVX for expression in *N. benthamiana* |
| BAX-Pt494-F | CCCATCGATAGACGAACAGAAGTGTGTATA | Clone CSEPs to PVX for expression in *N. benthamiana* |
| BAX-Pt494-R | TCCCCCGGGGTTGCTAAGATGGTAGCCTC | Clone CSEPs to PVX for expression in *N. benthamiana* |
| BAX-Pt3081-F | CCCATCGATGAAGGCCTAGGATGGAAG | Clone CSEPs to PVX for expression in *N. benthamiana* |
| BAX-Pt3081-R | TCCCCCGGGCGACTTCAAGAAATCGAGTA | Clone CSEPs to PVX for expression in *N. benthamiana* |
| BAX-Pt97002-F | CCCATCGATGATGATCCAGCTGGAAAAAC | Clone CSEPs to PVX for expression in *N. benthamiana* |
| BAX-Pt97002-R | TCCCCCGGGTTTGGTTGTAACACAAC | Clone CSEPs to PVX for expression in *N. benthamiana* |
| BAX-Pt15387-F | CCCATCGATGATGAAAAGGCCAGCCCAAA | Clone CSEPs to PVX for expression in *N. benthamiana* |
| BAX-Pt15387-R | TCCCCCGGGTGCACTAGTGAGGAGCA | Clone CSEPs to PVX for expression in *N. benthamiana* |
| BAX-Pt34354-F | CCCATCGATGATGCCGGGGCCCAGACGCC | Clone CSEPs to PVX for expression in *N. benthamiana* |
| BAX-Pt34354-R | TCCCCCGGGTGTACCAGTAGTGCCAGTAG | Clone CSEPs to PVX for expression in *N. benthamiana* |
| BAX-Pt88286-F | CCCATCGATTCTTCTGCCGAGCTCGCCGT | Clone CSEPs to PVX for expression in *N. benthamiana* |
| BAX-Pt88286-R | TCCCCCGGGTGCTTTGTTCTTGTCTT | Clone CSEPs to PVX for expression in *N. benthamiana* |
| BAX-Pt15525-F | CCCATCGATGCGGAATTGCAAACCAAATCT | Clone CSEPs to PVX for expression in *N. benthamiana* |
| BAX-Pt15525-R | TCCCCCGGGAGACCCAGCCGCGGGGGTTGT | Clone CSEPs to PVX for expression in *N. benthamiana* |
| BAX-Pt8502-F | CCCATCGATGCCCCGGAAGGCGTGAA | Clone CSEPs to PVX for expression in *N. benthamiana* |
| BAX-Pt8502-R | TCCCCCGGGGAGGATGCCACCGAGAAGTCC | Clone CSEPs to PVX for expression in *N. benthamiana* |
| BAX-Pt15546-F | CCCATCGATAATTGGGACCCCGCAACGGG | Clone CSEPs to PVX for expression in *N. benthamiana* |
| BAX-Pt15546-R | TCCCCCGGGCTAATCACTGTTGTCAGGAG | Clone CSEPs to PVX for expression in *N. benthamiana* |
| BAX-Pt20779-F | CCCATCGATACAGAGAACGATGAGCTTCC | Clone CSEPs to PVX for expression in *N. benthamiana* |
| BAX-Pt20779-R | TCCCCCGGGTTACGGTCCACTGCATCCTT | Clone CSEPs to PVX for expression in *N. benthamiana* |
| BAX-Pt8638-F | CCCATCGATAGTCTGAAGCTTGACTCCGTCC | Clone CSEPs to PVX for expression in *N. benthamiana* |
| BAX-Pt8638-R | TCCCCCGGGATTAGGCACGGGAGCGTAGTTC | Clone CSEPs to PVX for expression in *N. benthamiana* |
| BAX-Pt77192-F | CCCATCGATCTGCTAACGGGATTTGATCC | Clone CSEPs to PVX for expression in *N. benthamiana* |
| BAX-Pt77192-R | TCCCCCGGGACGCCTGGTGCGGGGGTAGTTC | Clone CSEPs to PVX for expression in *N. benthamiana* |
| BAX-Pt1625-F | CCCATCGATATGCTTCCACCATTAAAATT | Clone CSEPs to PVX for expression in *N. benthamiana* |
| BAX-Pt1625-R | TCCCCCGGGTCAAGTATGCTCTGCCCAAG | Clone CSEPs to PVX for expression in *N. benthamiana* |
| BAX-Pt23713-F | CCCATCGATAAAGCGCAAGTGTCGCGCTA | Clone CSEPs to PVX for expression in *N. benthamiana* |
| BAX-Pt23713-R | TCCCCCGGGGCCTCAAAGACTTCCACG | Clone CSEPs to PVX for expression in *N. benthamiana* |
| BAX-Pt36553-F | CCCATCGATAGGGATTATTCGCTACGAGG | Clone CSEPs to PVX for expression in *N. benthamiana* |
| BAX-Pt36553-R | TCCCCCGGGTTATAGGCCCGGGCATTCCT | Clone CSEPs to PVX for expression in *N. benthamiana* |
| BAX-Pt18222-F | CCCATCGATCTTTCCACGTGTGGGGACT | Clone CSEPs to PVX for expression in *N. benthamiana* |
| BAX-Pt18222-R | TCCCCCGGGAGCTGGCGGATACCTTGTTGCT | Clone CSEPs to PVX for expression in *N. benthamiana* |
| BAX-Pt36853-F | CCCATCGATATGATTTCCGTTTGTCACCA | Clone CSEPs to PVX for expression in *N. benthamiana* |
| BAX-Pt36853-R | TCCCCCGGGTTATTTCCAAACCGTGTCTG | Clone CSEPs to PVX for expression in *N. benthamiana* |
| BAX-Pt2567-F | CCCATCGATACACCGGGTTTGATGCCT | Clone CSEPs to PVX for expression in *N. benthamiana* |
| BAX-Pt2567-R | TCCCCCGGGTGCAATAGTTGTGTCGGAAGCG | Clone CSEPs to PVX for expression in *N. benthamiana* |
| BAX-Pt96482-F | CCCATCGATGACGATGCAGCCACCCCCAA | Clone CSEPs to PVX for expression in *N. benthamiana* |
| BAX-Pt96482-R | TCCCCCGGGTTGCGGGCTAGAGAGGAGCA | Clone CSEPs to PVX for expression in *N. benthamiana* |
| BAX-Pt29088-F | CCCATCGATGCCGCTGTGCTTCCGGTCAACG | Clone CSEPs to PVX for expression in *N. benthamiana* |
| BAX-Pt29088-R | TCCCCCGGGAGCGACTGCCACAGCGGGTG | Clone CSEPs to PVX for expression in *N. benthamiana* |
| BAX-Pt16552-F | CCCATCGATATGACACTGTGTCGAGTAATTCTTG | Clone CSEPs to PVX for expression in *N. benthamiana* |
| BAX-Pt16552-R | TCCCCCGGGATTGAGGATTTTCCTTGAT | Clone CSEPs to PVX for expression in *N. benthamiana* |
| BAX-Pt16-F | CCCATCGATATGGCTCGCATGGCTGAGCT | Clone CSEPs to PVX for expression in *N. benthamiana* |
| BAX-Pt16-R | TCCCCCGGGTGGGTACCACGCACTGCATT | Clone CSEPs to PVX for expression in *N. benthamiana* |
| BAX-Pt17-F | CCCATCGATATGTACCCGTACGTCGAGCT | Clone CSEPs to PVX for expression in *N. benthamiana* |
| BAX-Pt17-R | TCCCCCGGGCTACAGGGCGGAAAGAAGGG | Clone CSEPs to PVX for expression in *N. benthamiana* |
| BAX-Pt3372-F | CCCATCGATATGCACTTCGCTACCGTTCA | Clone CSEPs to PVX for expression in *N. benthamiana* |
| BAX-Pt3372-R | TCCCCCGGGCTATTCTGCAGAAGACTGTT | Clone CSEPs to PVX for expression in *N. benthamiana* |
| BAX-Pt14306-F | CCCATCGATATGCGCTTATCGGTCTATGT | Clone CSEPs to PVX for expression in *N. benthamiana* |
| BAX-Pt14306-R | TCCCCCGGGCTAGTGTTTTCGGCACTTTT | Clone CSEPs to PVX for expression in *N. benthamiana* |
| BAX-LBA | CAATCACAGTGTTGGCTTGC | For detection of BAX positive clones |
| BAX-LBB | GACCCTATGGGCTGTGTTG | For detection of BAX positive clones |

**Supplementary Table S2.** qRT-PCR primers

| Primer name | Primer 5'-3' | Purpose |
| --- | --- | --- |
| RT-Pt77192-F | GTGAATATCGCCGACCTGAT | qRT-PCR for validation of the expression patterns of CSEPs |
| RT-Pt77192-R | GAGCGCTCTTGCAGGATTT | qRT-PCR for validation of the expression patterns of CSEPs |
| RT-Pt5794-F | ATGCGATGGAATGATGGTGC | qRT-PCR for validation of the expression patterns of CSEPs |
| RT-Pt5794-R | TCGCGTTGTGTCATCTTTGT | qRT-PCR for validation of the expression patterns of CSEPs |
| RT-Pt34354-F | ATGAAAGGCGCAGCTACATT | qRT-PCR for validation of the expression patterns of CSEPs |
| RT-Pt34354-R | GCTACTGCTCCTTGGCATTC | qRT-PCR for validation of the expression patterns of CSEPs |
| RT-Pt23713-F | GAAAAATGCCACGACTGACC | qRT-PCR for validation of the expression patterns of CSEPs |
| RT-Pt23713-R | GACAATCCGGTCGAAAGGTA | qRT-PCR for validation of the expression patterns of CSEPs |
| RT-Pt16481-F | AATGGGACTCACAGCATTCC | qRT-PCR for validation of the expression patterns of CSEPs |
| RT-Pt16481-R | GAGACGCCGAAAGTATACGG | qRT-PCR for validation of the expression patterns of CSEPs |
| RT-Pt15525-F | AGGCAGTTACGAACCACCAC | qRT-PCR for validation of the expression patterns of CSEPs |
| RT-Pt15525-R | ACCGGATGTTGGAAGACAAC | qRT-PCR for validation of the expression patterns of CSEPs |
| RT-Pt1625-F | TGTGAAAGATCGGCAACAGA | qRT-PCR for validation of the expression patterns of CSEPs |
| RT-Pt1625-R | TGTGCTTAGTCGGATCGAGA | qRT-PCR for validation of the expression patterns of CSEPs |
| RT-Pt36553-F | CTTGCATTTCCTCAGCATCA | qRT-PCR for validation of the expression patterns of CSEPs |
| RT-Pt36553-R | CGGATCCGAGACTGCTTTAG | qRT-PCR for validation of the expression patterns of CSEPs |
| RT-Pt12858-F | CTACCAGGCCTTGTGGATGT | qRT-PCR for validation of the expression patterns of CSEPs |
| RT-Pt12858-R | GATATTCCCGCAAGCAAGTC | qRT-PCR for validation of the expression patterns of CSEPs |
| RT-Pt94682-F | GTTAAAGGCCTGCAGCAAAT | qRT-PCR for validation of the expression patterns of CSEPs |
| RT-Pt94682-R | CGGGCTAGAGAGGAGCATAA | qRT-PCR for validation of the expression patterns of CSEPs |
| RT-Pt18222-F | GCTCAGAGTAGTCGATGGCT | qRT-PCR for validation of the expression patterns of CSEPs |
| RT-Pt18222-R | GGATCTCCGCCAAGCTCTAT | qRT-PCR for validation of the expression patterns of CSEPs |
| RT-Pt29088-F | CATTCACCAAGCTCGACAGG | qRT-PCR for validation of the expression patterns of CSEPs |
| RT-Pt29088-R | CCATGTGGTCGAATTGGGTC | qRT-PCR for validation of the expression patterns of CSEPs |
| RT-Pt36853-F | TGTCAAAGGTTATGCGGCAG | qRT-PCR for validation of the expression patterns of CSEPs |
| RT-Pt36853-R | TTTCCAAACCGTGTCTGTGC | qRT-PCR for validation of the expression patterns of CSEPs |
| RT-Pt20779-F | CGGCGTGTTTACAACAGAGA | qRT-PCR for validation of the expression patterns of CSEPs |
| RT-Pt20779-R | CATCTCATACCCGGAACACC | qRT-PCR for validation of the expression patterns of CSEPs |
| RT-EF1-F | TCGTGTCGAAACCGGTACCATCAA | Reference gene of qRT-PCR |
| RT-EF1-R | AAACCAACGTTGTCACCTGGCAT | Reference gene of qRT-PCR |
